# *Calodromius bifasciatus* and other Carabidae on 26 oak-trunks in a nature reserve in the Netherlands

**DOI:** 10.3897/zookeys.100.1544

**Published:** 2011-05-20

**Authors:** Ron Felix, Paul van Wielink

**Affiliations:** 1 Hazelaarlaan 51, 5056 XP Berkel Enschot, The Netherlands; 2 Tobias Asserlaan 126, 5056 VD Berkel Enschot, The Netherlands

**Keywords:** arboricolous, Dromius s.l., *Laemostenus terricola*, phenology, spheres

## Abstract

The discovery of *Calodromius bifasciatus* in the nature reserve ‘De Kaaistoep’, the Netherlands, initiated research on this and related carabid beetles between 2000 and 2006. During this period we investigated the trunks of 26 Pedunculate oaks, mainly during nightly observations, to learn more about arboricolous carabid species. We observed more than 3000 specimens of 24 carabid species. The majority of these species were *Dromius* *s.l.*, however *Calodromius bifasciatus* dominated the dataset. Our data on phenology clearly show that *Calodromius bifasciatus* is mainly active in winter; it even copulates just above freezing point. Other interesting observations were made as well; for instance the presence of a small sphere at the end of the abdomen and their hiding behaviour at low temperatures. Subsequently, we obtained similar information about other tree dwelling carabid species. In this article we present an overview of all species observed on the trunks, after which we shall focus on the observations made on *Calodromius bifasciatus*.

## Introduction

A decade ago, the discovery of *Calodromius bifasciatus* (Dejean) on the trunk of a small Pedunculate oak (*Quercus robur*) near Tilburg (the Netherlands) was the start of a long term survey of tree trunks in that area. Until its discovery in the Netherlands, *Calodromius bifasciatus* was considered a Western Mediterranean species (Felix amd Van Wielink 2000). Because *Calodromius bifasciatus* turned out not to be uncommon in the area of discovery, research into the biology and ecology of the species was initiated.

A literature survey revealed that occurrence data from Northern France, Italy, Switzerland, Germany and Eastern Europe were based on very old records and could not be checked. Its presence and/or arrival in the Netherlands therefore seems difficult to relate to its currently known occurance. A source area for this species remains unknown. Although *Calodromius bifasciatus* is macropterous, flight observations are unknown. To obtain more insight into its dispersal behaviour, we placed flight interception traps (window traps) near and under trees where this carabid occurs, pitfall traps in the ground near the base of the trunk (but not beyond the outer range of the crown) but we never caught *Calodromius bifasciatus*. This was also the case with a frequently used light trap and a malaise trap in the vicinity.

Subsequently, we concentrated on the trunks themselves to learn more about the behaviour and life cycle of *Calodromius bifasciatus*. During this study we observed many more beetles and other tree-dwelling species (Van Wielink and Felix 2009a,b). Very little is known about the ecology of *Calodromius bifasciatus*, several publications only mention that the species has been found under the bark of dead trees. According to other publications it is a corticolous species (inhabiting bark), with a tendency to lapidicol (living under stones) (Aguiar and Serrano 1995, Ortuño and Torribio 1996). In Algeria it was found in the galleries of *Scolytes* species in branches of cedars and oaks (Mehenni and Bosmans 1994).

In this paper we present information on the biology and ecology of *Calodromius bifasciatus*, as could be gathered by observing and collecting the species from 26 Pendunculate oak trees. Additionally, we provide some results on other observed carabid species of the same tree trunks.

## Site description, material and methods

### De Kaaistoep

The nature restoration area ‘De Kaaistoep’ lies immediately west of Tilburg in the south of the Netherlands. It is a former agricultural area, belonging to a waterworks company. The actual research site consists of open arable grasslands on poor sandy soil. This open area is bordered by woodland in the west, north and east. In the area itself there are three large and two small artificial pools, a brook and some patches or rows of deciduous trees and shrubs. Almost in the middle of these grasslands there are two rows of Pedunculate oaks, the trunks of which were investigated. One short row (A) runs from north-north-west to south-south-east and numbers seven oaks. Another, longer, row (B) runs from south-west to north-east and numbers 19 oaks. Some of the trees in row B stand alone, this means that their crowns do not touch other trees. Most of the oaks in row B are bigger and have lower branches than the oaks in row A. The ground around row B is covered with shoots of European elder (*Sambucus nigra*) and American black cherry (*Prunus serotina*), grasses and dead twigs and branches of the trees. Many rabbit holes surround row B. The trunks of row A are more exposed to the sun and wind than those of row B. In row A the ground around the trunks is only sparsely vegetated with short grasses, a few mosses and only few tree branches lie on the ground. All trunks are covered by lichens and algae and at the base of the trunks mosses are present. The oaks in row A are much more densely covered by lichens than those of row B. All oaks are healthy and undamaged. They stand at various distances from each other, are 15–22 m tall, bear a crown of 10–20 m in diameter and have a trunk girth of 90–230 cm. More details on ‘De Kaaistoep’ can be found in Felix and Van Wielink (2008).

### Sampling the tree trunks

**Figure 1.**
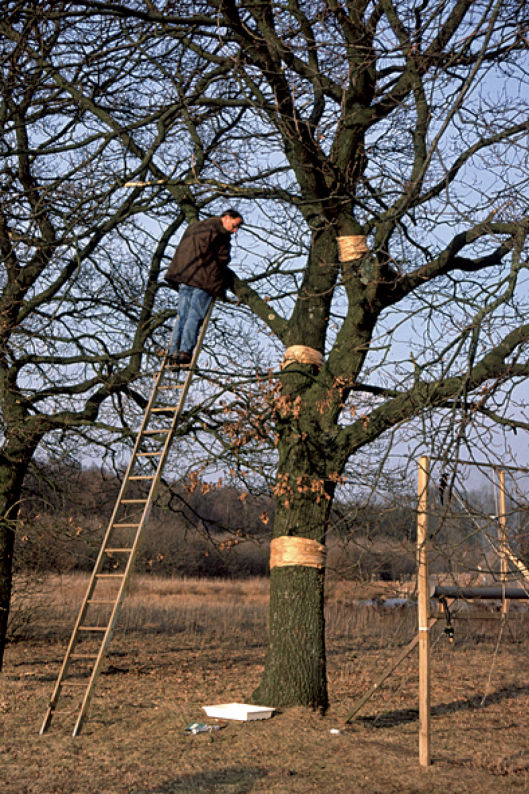
Paper bands at several heights. Photo: Paul van Wielink

We used a non-standard method to collect carabid beetles from the tree trunks: ‘wrapped paper bands’ (Fig. 1). The bands consisted of packing paper, longitudinally rumpled and wrapped around the trunk. They were put on two oak trees (on A5, i.e., the fifth tree from the north in row A; and on B6, i.e., the sixth oak from the west of row B). The bands were placed at about 1.6 m above ground level and were operational for four years. Later we installed additional bands around branches of tree A5 at various heights (4, 6 and 7 m high and close to the trunk, and one at 6 m height but at 4 m distance from the trunk). These additional bands were operational for three years. Every 6–8 weeks the bands were inspected by shedding the paper bit by bit over a white plastic tray. While removing the paper bands the bark underneath was carefully inspected and carabid beetles were identified and afterwards often released on the trunk they originated from.

### Monitoring the tree trunks at night

During more than six years, we monitored all 26 oaks from the base up to about 3 m height, 144 times at night. For more than two years within that period, the inspections were carried out nearly every week always on the same day (104 times). We started 1 to 6 hours after sunset; early in summer and in relatively late winter. Each visit took 35 to 90 minutes, depending on the number of beetles found. The trunks were illuminated by torch and we counted and noted the carabid beetles and their behaviour. For *Dromius* *s.l.* species (which includes Dromius, Calodromius, Paradromius and Philorhizus species), we noted the height and direction on the trunk, observations on their mating and other behaviour, as well as the presence of spheres on the tip of their abdomen (apex). Weather conditions (temperature, direction and strength of the wind, humidity, presence of fog, etc.) were noted as well. The light of the torches used was too bright and disturbed the beetles. They immediately tried to seek a hiding place or even dropped to the ground, so we switched to using LED lights. Again, almost all carabid beetles were released after identification. The circumference of each tree was measured at 1.60 m height, as well as the depth of the clefts in the bark at that height. A more detailed description of these methods can be found in Felix and Van Wielink (2008).

## Results

### Carabid beetles on tree trunks

**Figure 2.**
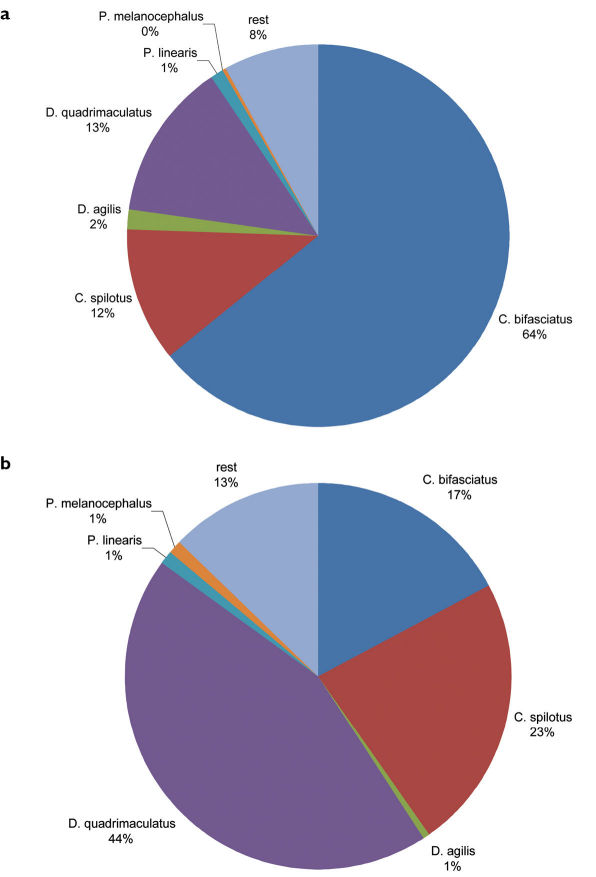
Relative abundance ofCarabidaeduring weekly observations at night (**a**) and in the bands (**b**). (**a**) Of the 1903 Carabidaeobserved weekly at night, *Dromius* *s.l.* (the *Dromius*, *Calodromius*, *Paradromius* and *Philorhizus* species mentioned in the graph)accounts for 92% and *Calodromius bifasciatus alone* for 64%. (**b**) Of the 373Carabidaeobserved in the bands, *Dromius* *s.l.* accounts for 87% and *Calodromius bifasciatus* for 17%.

In total, we observed 3069 specimens of 24 carabids beetle species (Table 1). Of all carabid beetles found in the bands, 87% were *Dromius* *s.l.* and 17% *Calodromius bifasciatus* (Fig. 2). Of all carabid species noted during nightly observations, 92% were *Dromius* *s.l.* and 64% *Calodromius bifasciatus* (Fig. 2).

**Table 1.** Survey of species and numbers of Carabidae observed at nightly inspections and in or behind bands on oak trees.

*Species*	*Nightly inspection*	*In/behind bands*
*Carabus problematicus* Herbst, 1786	18	2
*Carabus nemoralis* Müller, 1764	5	-
*Leistus rufomarginatus* (Duftschmid, 1812)	4	-
*Leistus spinibarbis* (Fabricius, 1775)	44	1
*Leistus ferrugineus* (Linnaeus, 1758)	8	1
*Nebria brevicollis* (Fabricius, 1792)	2	3
*Nebria salina* Fairmaire & Laboulbene, 1854	-	9
*Nebria brevicollis/salina*	13	-
*Notiophilus rufipes* Curtis, 1829 (larf)	1	-
*Trechus obtusus* Erichson, 1837	1	-
*Bembidion tetracolum* Say, 1823	1	-
*Pterostichus niger* (Schaller, 1783)	1	2
*Calathus melanocephalus* (Linnaeus, 1758)	-	1
*Calathus rotundicollis* Dejean, 1828	1	-
*Laemostenus terricola* (Herbst, 1784)	77	27
*Limodromus assimilis* (Paykull, 1790)	1	-
*Agonum thoreyi* Dejean, 1828	1	-
*Bradycellus harpalinus* (Serville, 1821)	9	-
*Bradycellus verbasci* (Duftschmid, 1812)	1	-
*Dromius agilis* (Fabricius, 1787)	41	2
*Dromius quadrimaculatus* (Linnaeus, 1758)	377	165
*Paradromius linearis* (Olivier, 1795)	38	4
*Calodromius bifasciatus* (Dejean, 1825)	1654	64
*Calodromius spilotus* (Illiger, 1798)	378	86
*Philorhizus melanocephalus* (Dejean, 1825)	10	4
larvae undet	8	4
Total number	2694	375

We observed six species of *Dromius* *s.l.* in and underneath the bands and during the nightly observations: 1718 *Calodromius bifasciatus* specimens (1654 at night and 64 in the bands), 542 *Dromius quadrimaculatus* (377 at night and 165 in the bands), 464 *Calodromius spilotus* (378 at night and 86 in the bands), 43 *Dromius agilis* (41 at night and 2 in the bands), 42 *Paradromius linearis* (38 at night and 4 in the bands) and 14 *Philorhizus melanocephalus* (10 at night and 4 in the bands). It must be noted that these numbers give no reliable indication of population size. Because we seldom collected specimens, many specimens were probably counted several times.

On some trees we always saw more *Calodromius bifasciatus* than on other trees. We counted the number of specimens we saw on every tree. We computed the amount of square meters of a trunk up to 2.5 m of the tree and the average number of specimens per square meter of that part of the trunk, and there was no relation between the circumference of the tree and the number of specimens. We also counted every *Calodromius spilotus* and *Dromius quadrimaculatus* observed. There was no correlation between the numbers of the three carabid species on the separate trees. For results concerning these observations we refer to a previous publication (Felix & Van Wielink 2008). On some trees we saw many of ants (Formicidae) or isopods (Isopoda), and some trees carried more algae, lichens or mosses than others. We never quantified these phenomena, but we have gained the impression that there is also no relation between the abundance of the mentioned species and *Calodromius bifasciatus*.

The four most abundant species *Dromius* *s.l.*, *Calodromius bifasciatus*, *Calodromius spilotus* and *Dromius quadrimaculatus* were also present in the band on a branch at 6 m height and at 4 m from the main trunk but in far lower quantities than in the other bands. All specimens of *Dromius agilis* were found in row B. Perhaps the sheltered position of the trunks in this row explains this observation.

### Phenology of *Calodromius bifasciatus* and *Dromius* *s.l.* species

*Calodromius bifasciatus* was active on the bark at an air temperature between -3.5 and 17°C, and mostly in the range 4–8°C. The maximum number of specimens we observed on one single evening was 85 on the 20th November and the 27th of December 2003. The temperatures were 8°C and 6°C respectively, the wind was southwest, strength 4 and 3. During both evenings the atmosphere was humid without rain or wet trunks. Hannig et al. (2006) found similar weather preferences for *Calodromius bifasciatus*. The number of *Calodromius bifasciatus* specimens per tree varied considerably: the total number of specimens varied from 20 to 169, with three trees with more than 120 observed specimens. Although the exact numbers are different when taking the area of inspection into consideration (i.e., individuals/m2 trunk varied between 4.7 and 34.5), the same trees had the highest numbers. We observed one tree several times during one night; even the presence on one oak in one night at several times in time varied substantially. Also the position on the bark varies substantially (for details see Felix & Van Wielink 2008).

**Figure 3.**
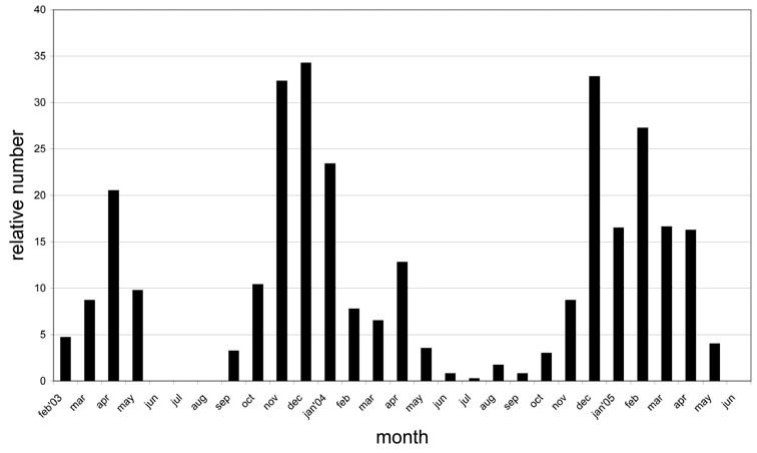
Phenology of *Calodromius bifasciatus*: relative presence in 29 consecutive months. Relative presence: the number of beetles per month divided by the number of nightly observations during that month (the number of nightly observations varied between 2 and 4 per month).

**Figure 4.**
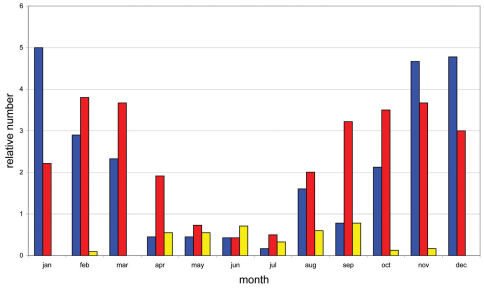
Phenology of *Calodromius spilotus*, *Dromius quadrimaculatus* and *Dromius agilis*: relative average presence per month. Relative presence: see Fig. 3. Blue: *Calodromius spilotus*, Red: *Dromius quadrimaculatus*, Yellow: *Dromius agilis*.

Based on weekly observations at night on the lower 3 m of the trunks during two years we can present the phenology of *Calodromius bifasciatus* (Fig. 3). Its main activity takes place in winter: observation periods November 2003 to January 2004 and December 2004 to February 2005. In summer, this species was almost absent. *Dromius quadrimaculatus* and *Calodromius spilotus* have their optimum from September to March (Fig. 4). *Dromius agilis* was seen in far lower numbers, and practically only from April to September (Fig. 4).

### Observations on behaviour and biology of *Calodromius bifasciatus*

During the 144 nightly observations, we noted 63 copula in 1654 *Calodromius bifasciatus* of which there were 46 copula amongst 1219 specimens during the 104 weekly observations. Copula were seen during the whole activity period from October to April, and at temperature ranges of -1 to 17°C. During the evening, eight copula were seen; 21% of the observed specimens. The temperature that evening was 8°C and humidity was high. Also *Calodromius spilotus* was seen copulating in winter.

We never found larvae, neither on the trunks at night, nor in the bands. However we found two freshly emerged specimens of *Calodromius bifasciatus* on a trunk on the 24th August 2001, indicating recent pupation at this time and location.

**Figure 5.**
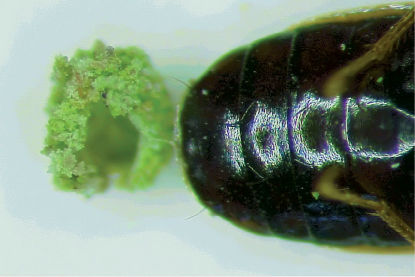
Abdomen with sternites of *Calodromius bifasciatus* with a sphere. Note the granulate outside of the sphere. Photo: Ron Felix.

Observing *Calodromius bifasciatus*, we noticed ‘spheres’ on the abdomen of females (Fig. 5). These spheres were different in size, from about 0.3 to 1.0 mm. Their outside is granulated with lichens or algae and the inner side is very smooth. These spheres are probably egg-cases (see discussion). We collected a few females with spheres and almost always they dropped these structures rapidly. We noted 69 females with a smaller or greater sphere during the 144 nightly observations. In the 104 weekly observations there were 60 of them, about 5% of the observed specimens. The spheres were almost exclusively seen from November to May. A few times we even saw matings while the female was bearing a sphere. The spheres were seen during nights when the temperature was between 3 and 15°C, so within the normal range of activity and copula. We also noted behaviour that indicated how *Calodromius bifasciatus* makes these spheres. We found *Calodromius bifasciatus* specimens biting algae or lichens, then stepping forward and rubbing the tip of the abdomen over the spot where they had just bitten. While biting, the abdomen was directed upwards and the hind legs were stretched. While rubbing their abdomen against the lichens, the posture was reverse. Sometimes the specimens had a sphere on their abdomen, sometimes they did not. When we saw this behaviour, the temperatures ranged from 3 to 13°C and air humidity was usually high.

## Discussion and conclusions

### Carabid beetles on tree trunks

*Dromius* *s.l.* species were by far the most abundant Carabidae on the trunks. In addition, 23 other species of Carabidae were observed, most of them in very low numbers (Table 1). Some genera were often found on the trunk, whether in sight at night, or in/underneath the paper bands: *Carabus* *s.l.* (especially *Carabus problematicus*) *Leistus s.str*. (especially *Leistus spinibarbis*), *Nebria s.str.* and *Laemostenus terricola*. With some of these species, it was previously unknown that they climbed trees. For example, *Paradromius linearis* and *Philorhizus melanocephalus* were known as ‘strictly ground dwelling’ (Turin 2000). One of the most abundant other Carabidae seen on the trunks (and almost exclusively on those of row B) was *Laemostenus terricola*. In the Netherlands this species is known especially from sandy, hilly, warm regions. In the northern parts of its distribution area it is often found in cellars, but also in rabbit holes and underneath stones, in woods and heathlands. It is not clear what the relation is between its presence in woods and heaths and the presence of mammal holes. Several times we saw specimens walking from the deep of a rabbit hole, close to the trunk of an oak, towards the entrance. As soon as they were illuminated, they ran back, as in panic, into the darkness of the rabbit hole. We saw 77 specimens at night, especially in summer, sometimes high up on a tree trunk. We also noticed individuals on the ground between tree trunks and nearby rabbit holes. The times we saw them were 1.5–2.5 hours after sunset at temperatures between 10 and 18°C. We assume that this species rests in rabbit holes and forages in the trees.

### Biology of *Calodromius bifasciatus* and other *Dromius* *s.l.* species

*Dromius* *s.l.* species are flat (but relatively broad) and are built to hide in very narrow places. It probably moves all over the trees when it is dark and during daytime it stays in the lower parts, where hiding is easier. Trunks with (at least partially) a structure of many fine and narrow clefts (in the lower parts) would be more appreciated by *Calodromius bifasciatus* and other tree-living *Dromius* *s.l.* species than trunks with deep, but open and wide clefts. Scheffler (1997) found that *Calodromius spilotus* prefers places in which it experiences pressure: aggregation experiments showed that more specimens crawl underneath flat filter paper on the bottom of Petri dishes, than under folded paper. This also explains why we found more specimens underneath than within the paperbands.

*Calodromius bifasciatus* seems to avoid contact with the ground. We hardly ever found them in pitfall traps that were placed in the close surroundings of the investigated trees and we did not find them in the soil after digging at the foot of the tree either. Once we observed an agglomeration at the base of a tree. This agglomeration was observed during a very cold, freezing night, and was located about 1 cm below  the soil surface, but it was still on the bark. On the other hand, Van Malderen (2007) mentions that *Calodromius bifasciatus* can be found by sifting leaves and dead wood underneath oaks and poplars.

The common opinion seems to be that *Dromius* *s.l.* species are not seen on the lower parts of the tree in summer, because they are high up in the crown (Scheffler 1997, Irmler 1998, Simon 2001, Hannig et al. 2006). Temperatures should be lower there and humidity higher and there should be a greater availability of food in summer. Irmler (1998) installed window traps in forests at different heights and found *Dromius agilis* and *Dromius quadrimaculatus* more often in higher window traps than in lower ones in spring, summer and autumn. However, this only means that these species fly at these heights, but this does not mean that they stay high up in the trees in summer. Maybe these species need a specific height to fly away. In contrast to these studies, we did find *Dromius* *s.l.* species also in summer, in lower parts of trees. Our impression is that in summer clefts in the trunks, especially on shaded sides or on the north side at the bottom 2–3 m, are humid and cool too. Therefore the availability of food should be sufficient on the lower parts of the trunks as well. We suggest that the niche of *Calodromius bifasciatus* is determined by the presence of suitable clefts. In the crown and thinner branches clefts are hardly to be found. When threatened or disturbed by light, *Calodromius bifasciatus* immediately seeks shelter in the clefts of the trunk. These shelter opportunities seem harder to find high up in the trees where trunk and branches have no clefts. Furthermore, our bands around the trunks and branches provided no indication for migration upward to the tree crown in summer. There is no direct evidence for the actual presence of *Calodromius bifasciatus* or other *Dromius* *s.l.* species high up in the tree crown in summer. The absence of *Calodromius bifasciatus*, *Calodromius spilotus* and to a lesser extend *Dromius quadrimaculatus* could simply mean that only a few survive the warmer part of the year, if any. We assume that most adults of *Calodromius bifasciatus* die before summer.

### *Calodromius bifasciatus* reproduction

The behaviour of biting algae and sphere building has not been recorded before in Europe. Will (1998) extensively describes exactly the same behaviour prior to oviposition of *Dromius piceus* (Dejean) in Ithaca, New York, USA. Casale et al. (1996) also mention ‘egg-cases’ for *Dromius meridionalis* and *Dromius quadrimaculatus*. We did not find eggs in the spheres of *Dromius* *s.l.* species; all were empty. Perhaps the egg(s) is inserted into the sphere just before it is dropped. In this respect the spheres should probably be named ‘pre-ootheca’. We noticed spheres in almost every month, but predominantly in winter. Depending on the developmental time of the embryo and possible dormancy of the young larva, larvae could be active from early winter and adults may subsequently appear from August to late summer. This is in line with the discovery of two freshly emerged adults in August. Although we never found larvae, we assume that they live on the trunks as well and not on or in the ground. This assumption is also based on the observations of Casale et al. (1996) of *Dromius meridionalis* larvae on broad-leaved lime trees (*Tilia platyphyllos*) in a busy street where the ground surface was paved up to the trunk.

During the nightly observations many copula of *Calodromius bifasciatus* were seen during the whole activity period from October to April, during nights with temperatures ranging between -1 to 17 °C. Nightly observations on tree trunks, especially in autumn, winter and spring, can thus be very rewarding in observing ecological phenomena of corticolous Carabidae.
